# DL0410 Ameliorates Memory and Cognitive Impairments Induced by Scopolamine via Increasing Cholinergic Neurotransmission in Mice

**DOI:** 10.3390/molecules22030410

**Published:** 2017-03-06

**Authors:** Wenwen Lian, Jiansong Fang, Lvjie Xu, Wei Zhou, De Kang, Wandi Xiong, Hao Jia, Ai-Lin Liu, Guan-Hua Du

**Affiliations:** 1 Institute of Materia Medica, Chinese Academy of Medical Sciences and Peking Union Medical College, Xian Nong Tan Street, Beijing 100050, China; lianwenwen1989@imm.ac.cn (W.L.); fangjiansong0815@163.com (J.F.); xulvjie@imm.ac.cn (L.X.); zhouwei@imm.ac.cn (W.Z.); kangde@imm.ac.cn (D.K.); xiongwandi@imm.ac.cn (W.X.); jhaoer@imm.ac.cn (H.J.); 2 Beijing Key Laboratory of Drug Target Research and Drug Screening, Chinese Academy of Medical Sciences and Peking Union Medical College, Beijing 100050, China; 3 State Key Laboratory of Bioactive Substance and Function of Natural Medicines, Chinese Academy of Medical Sciences and Peking Union Medical College, Beijing 100050, China

**Keywords:** Alzheimer’s disease, DL0410, AChE inhibitor, BuChE inhibitor, ACh

## Abstract

Deficiency of the cholinergic system is thought to play a vital role in cognitive impairment of dementia. DL0410 was discovered as a dual inhibitor of acetylcholinesterase (AChE) and butyrylcholinestease (BuChE), with potent efficiency in in-vitro experiments, but its in vivo effect on the cholinergic model has not been evaluated, and its action mechanism has also not been illustrated. In the present study, the capability of DL0410 in ameliorating the amnesia induced by scopolamine was investigated, and its effect on the cholinergic system in the hippocampus and its binding mode in the active site of AChE was also explored. Mice were administrated DL0410 (3 mg/kg, 10 mg/kg, and 30 mg/kg), and mice treated with donepezil were used as a positive control. The Morris water maze, escape learning task, and passive avoidance task were used as behavioral tests. The test results indicated that DL0410 could significantly improve the learning and memory impairments induced by scopolamine, with 10 mg/kg performing best. Further, DL0410 inhibited the AChE activity and increased acetylcholine (ACh) levels in a dose-dependent manner, and interacted with the active site of AChE in a similar manner as donepezil. However, no difference in the activity of BuChE was found in this study. All of the evidence indicated that its AChE inhibition is an important mechanism in the anti-amnesia effect. In conclusion, DL0410 could be an effective therapeutic drug for the treatment of dementia, especially Alzheimer’s disease.

## 1. Introduction

Alzheimer’s disease (AD), the most common form of dementia in the aging population, is a progressive neurodegenerative disorder. Clinically, it is characterized by the progressive loss of learning and cognitive functions, as well as the impairment of memory [1]. There are three major pathological hallmarks, including the deposition of amyloid plaques, abnormal phosphorylation of tau protein, and the loss of neurons [2]. Although the etiology of AD is still not clear, the dysfunction of the cholinergic system is thought to play an important role in cognitive impairment [3,4]. The deficiency of acetylcholine (ACh), which results from the function loss of the cholinergic system or the loss of cholinergic neurons in the hippocampus and cortex, appears to contribute significantly to the decline in cognitive function, including the encoding and retrieval of information [5]. Thus, increasing endogenous ACh or activating ACh receptors has been explored as a method to treat dementing diseases, especially AD. Among these efforts, inhibition of acetylcholinesterase (AChE), which increases the accumulation of ACh in the synaptic cleft, was proved to be efficacious in improving cognitive function, and is currently the main therapeutic strategy for AD patients [6].

**Figure 1:**
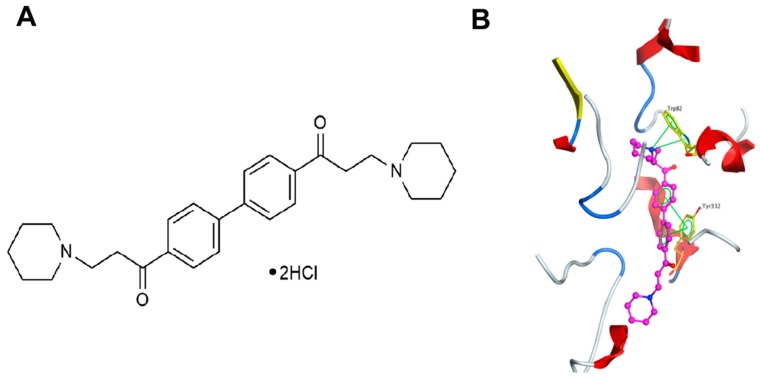
The chemical structure of DL0410 (**A**) and DL0410 in the active site of butyrylcholinestease (BuChE) (PDBID: 4BDS). DL0410 is displayed in the ball-stick model with carbon in purple, and the binding residues are shown in the stick model with carbon in yellow (**B**). Nitrogen is shown in blue and oxygen is in red.

DL0410 (Figure 1A) is a potent AChE inhibitor with IC_50_ = 0.29 μM, which is about twice as high than that of donepezil (IC_50_ = 0.085 μM). However, its inhibition of butyrylcholinestease (BuChE) was further discovered by machine learning, with IC_50_ = 3.96 μM, which is 10 times lower than that of donepezil [7,8]. In addition, the interaction between DL0410 and BuChE was shown in Figure 1B, which means that a cation-π and π-π interaction were made between DL0410 and BuChE. This is an advantage of DL0410 in comparison with donepezil. ACh could be hydrolyzed by two kinds of cholinesterase, AChE and BuChE. The former, expressed in neurons, is the dominantly-involved enzyme, and the latter only plays a supplementary role in the degradation of ACh. However, in AD patients, an increase of BuChE is found when AChE is lost [9]. Therefore, BuChE is a promising target for AD therapy [10,11]. As a dual inhibitor of AChE and BuChE, the in vivo effect of DL0410 has been verified in a series of AD-related mouse models, including Aβ_1-42_-induced amnesia in mice [8] and APP/PS1 transgenic mice [12]. These results indicated that DL0410 could effectively improve the learning process and cognitive impairments. DL0410, a potent AChE inhibitor in vitro, deserves more studies on its in vivo effects on the cholinergic transmission in a specific model with determinate modification in the cholinergic system. However, its in vivo effect on the cholinergic model has not been evaluated, and its action mechanism in the cholinergic system has also not been illustrated.

Scopolamine is a non-selective antagonist of the muscarinic acetylcholine receptor (mAChR). It could induce neuronal injury by blocking the muscarinic receptor and increasing the activity of AChE. Finally, it results in the deficit of ACh in the synaptic cleft, the blockage of signal transduction, and the failure of memory acquisition. Scopolamine-induced mice models with learning and memory impairments have been considered as acetylcholine system impairments and employed extensively to evaluate the memory-enhancing effects of new drugs [13].

In the present study, the effect of DL0410 on scopolamine-induced learning and memory impairments in mice was investigated by a series of behavior tests. In order to verify the involvement of cholinesterase inhibition in the molecular mechanism of DL0410, the ACh level and the activity of AChE and BuChE were also examined in the hippocampus. In addition, molecular docking was employed to explore the interaction and binding mode between DL0410 and cholinesterase.

## 2. Results

### 2.1. The Effect of DL0410 on the Locomotor Activity

**Figure 2:**
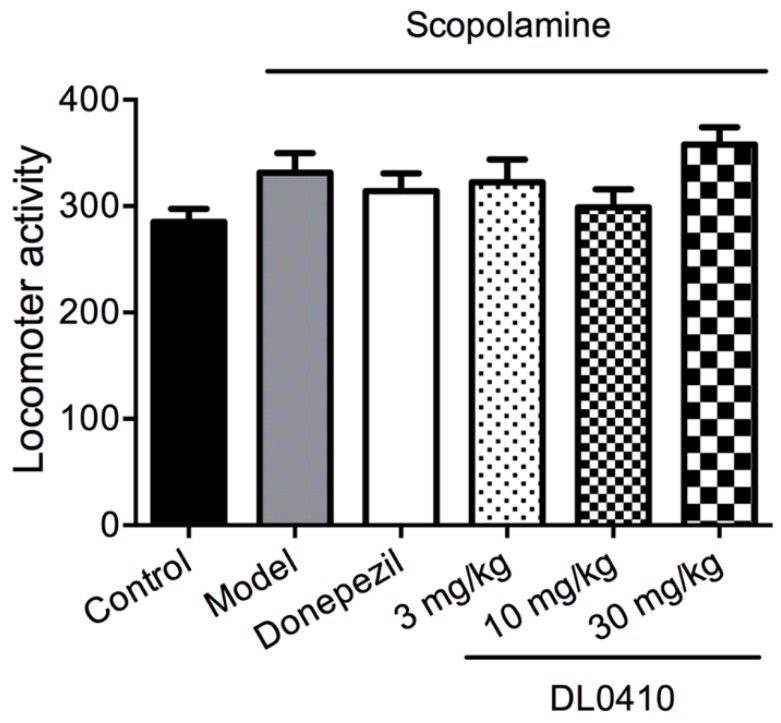
The effect of DL0410 on locomotor activity. There were no significant differences among groups. Data are expressed as mean ± SEM (*n* = 12).

Since all behavior tests were carried out after the administration of scopolamine and drugs (donepezil and DL0410), locomotor activity was evaluated to exclude the potential effects of scopolamine and drugs on sensory and motor functions. As shown in Figure 2, compared with the control group, administration of scopolamine in the presence or absence of drugs (donepezil and DL0410) had no effects on the locomotor activity of mice (*p* > 0.05). Therefore, the following behavior tests could be used to evaluated the ability of learning and cognition of mice.

### 2.2. DL0410 Improved the Spatial Memory Impairments of Mice Induced by Scopolamine in the Morris Water Maze

**Figure 3:**
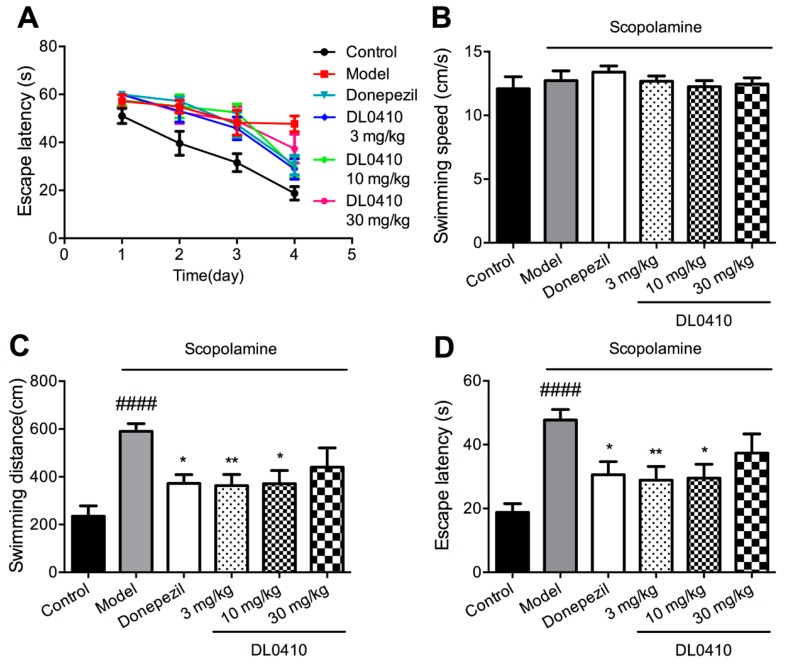
The improvement of DL0410 on the impaired spatial memory induced by scopolamine in the navigation trial of the Morris water maze. Data were expressed as mean ± SEM (*n* = 12). (**A**) The mean escape latency in the navigation trials; (**B**) The mean swimming speed of mice on the fourth day; (**C**) The swimming distance of mice to find the hidden platform on the fourth day; (**D**) The escape latency of mice to find the hidden platform on the fourth day. ^####^ *p* < 0.0001 vs. the control group; * *p* < 0.05 and ** *p* < 0.01 vs. the model group.

In the navigation trials, the escape latency of all groups shortened as the training times increased. As shown in Figure 3A, the escape latency of the first day was almost the same among all groups, which implied the same beginning of spatial memory of mice in all groups. As the training proceeded, the escape latency of all group decreased at different rates; that of the control group decreased faster than the other five groups treated with scopolamine. The performance on the fourth day was used as the final result of the navigation trial. There were no significant differences in swimming speed among all groups, as shown in Figure 3B. Compared with the control group, the escape latency and swimming distance of the model group were notably increased (*p* < 0.0001 for both). Compared with the model group, DL0410 (3 mg/kg and 10 mg/kg) could significantly reduce swimming distance and escape latency of mice (*p* < 0.01 for escape latency and swimming distance of DL0410 at 3 mg/kg; *p* < 0.05 for escape latency and swimming distance of DL0410 at 10 mg/kg). Donepezil could remarkably decrease the escape latency of mice (*p* < 0.05). The detailed information is shown in Figure 3C,D. Therefore, scopolamine could interfere with learning processes, and treatment with DL0410 effectively improved the ability of learning and memory.

**Figure 4:**
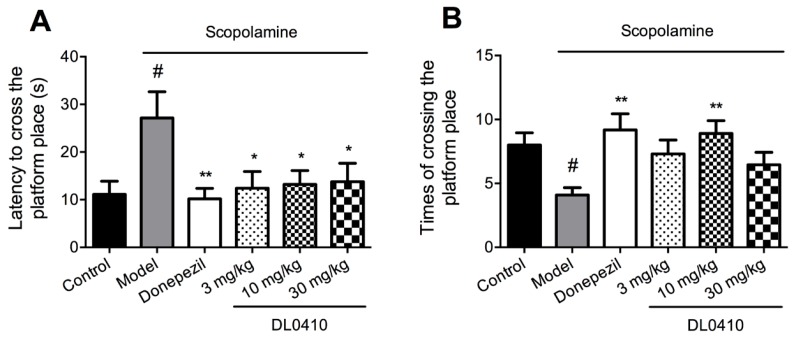
The improvement of DL0410 on the impaired spatial memory induced by scopolamine in the probe trial of the Morris water maze. Data were expressed as mean ± SEM (*n* = 12). (**A**) The latency to cross the platform position in the probe trial; (**B**) The times of crossing the platform position in the probe trial. ^#^ *p* < 0.05 vs. the control group; * *p* < 0.05 and ** *p* < 0.01 vs. the model group.

In the probe trial, the platform was removed from the maze, and mice were allowed to swim freely for 120 s. Latency to cross the platform position and times of crossing the platform position were recorded to assess the retention of spatial memory. As shown in Figure 4A, mice in the model group had a longer latency to firstly cross the position of the platform than those in the control group (*p* < 0.05). Mice treated with DL0410 and donepezil exhibited a notably shorter latency than those in the model group (*p* < 0.05 for DL0410 at 3 mg/kg, 10 mg/kg, 30 mg/kg; *p* < 0.01 for donepezil). Figure 4B revealed that the times of crossing the platform of the model group were remarkably decreased than those of the control group (*p* < 0.05). Mice administrated with DL0410 3 mg/kg and donepezil could significantly increase times of crossing the platform in comparison with the model group (*p* < 0.01 for both). These findings indicated that DL0410 could ameliorate the deficits of spatial memory induced by scopolamine.

### 2.3. DL0410 Ameliorated Memory Impairment Induced by Scopolamine in the Passive Avoidance Task

**Figure 5:**
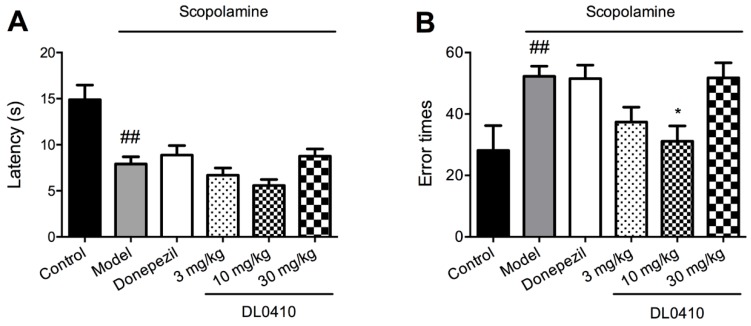
The effect of DL0410 on the passive avoidance task. Data were expressed as mean ± SEM (*n* = 12). (**A**) The latency that mice took to firstly step into black chamber in the retention test. Scopolamine could induce mice to step into the black chamber with shorter latency, and donepezil and DL0410 showed the tendency to extend the latency; (**B**) The error times of mice stepping into black chamber in the retention test. Scopolamine could induce mice to step more times into the black chamber, and DL0410 could reduce the times of mice stepping into the black chamber. ^##^ *p* < 0.01 vs. the control group; * *p* < 0.05 vs. the model group.

The step-through test is a kind of passive avoidance task, in which latency and error times were the two main indices. In the acquisition phase, there were no statistical differences in latency and error times among all groups (data not shown). As shown in Figure 5, in the retention phase, the latency of the model group was significantly lower than that of the control group, while DL0410 (3 mg/kg and 10 mg/kg) and donepezil slightly increased the latency compared with the model group. In terms of error times, mice in the model group stepped into the black chamber more times than the control group (*p* < 0.01), and DL0410 (10 mg/kg) could significantly decrease error times compared with the model group (*p* < 0.05).

### 2.4. DL0410 Ameliorated Memory Deficit Induced by Scopolamine in the Escape Learning Task

**Figure 6:**
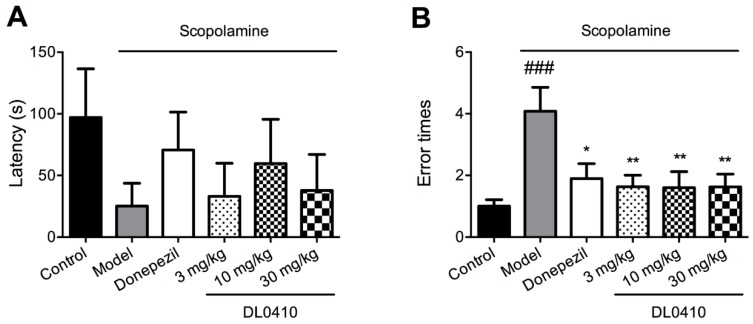
The effect of DL0410 on the escape learning task. Data were expressed as mean ± SEM (*n* = 12). (**A**) The latency that mice took to firstly jump to the metal floor in the retention test. Scopolamine could induce mice to step down to the metal floor with shorter latency, and donepezil and DL0410 showed the tendency to extend the latency; (**B**) The error times of mice jump to the metal floor in the retention test. Scopolamine could induce mice to step more times down to the metal floor, and DL0410 could reduce the times of mice stepping down to the metal floor. ^###^ *p* < 0.001 vs. the control group; * *p* < 0.05 and ** *p* < 0.01 vs. the model group.

The step-down test is a kind of escape learning task. The results of the escape learning task are shown in Figure 6. In the acquisition phase, there were no significant differences in the latency and error times (data not shown). However, in the retention phase, the latency of the model group was shorter than that of the control group, and DL0410, as well as donepezil, could increase the latency. The error times of the model group were significantly greater than the control group (*p* < 0.001), and DL0410 (3 mg/kg, 10 mg/kg, and 30 mg/kg), as well as donepezil, could decrease the error times (*p* < 0.05 for donepezil; *p* < 0.01 for DL0410 at 3 mg/kg, 10 mg/kg, and 30 mg/kg).

### 2.5. DL0410 Increased ACh Levels by the Inhibition of AChE in the Hippocampus but not BuChE

**Figure 7:**
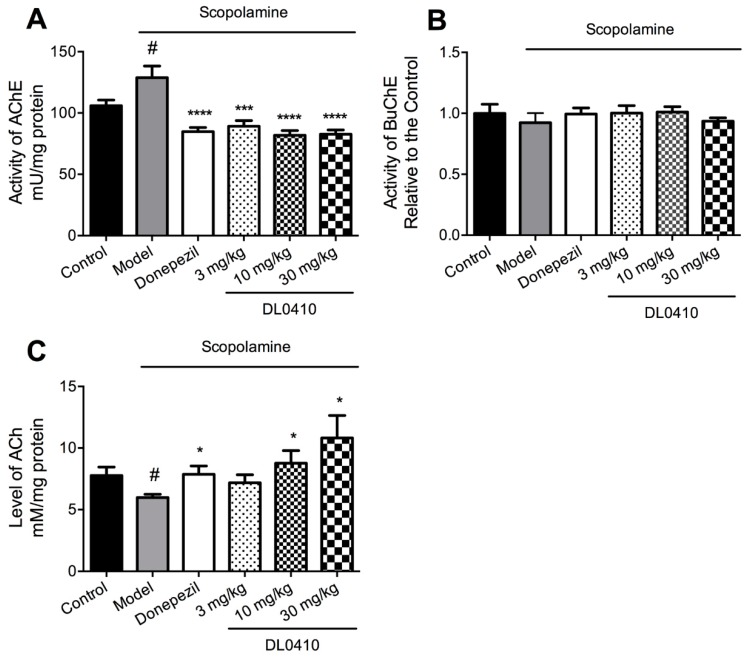
The effect of DL0410 on the activity of acetylcholinesterase (AChE) and BuChE and acetylcholine (ACh) levels in the hippocampus. Data are expressed as mean ± SEM (*n* = 6). (**A**) The activity of AChE in the hippocampus; (**B**) The activity of BuChE in the hippocampus; (**C**) The ACh level in the hippocampus. ^#^ *p* < 0.05 vs. the control group; * *p* < 0.05, *** *p* < 0.001 and **** *p* < 0.0001 vs. the model group.

In this study, the activity of AChE and BuChE and Ach levels in the hippocampus were determined. As shown in Figure 7A, the AChE activity of the model group was significantly higher than that of the control group, and DL0410 (1 mg/kg, 3 mg/kg, 10 mg/kg) notably reduced the increased AChE activity induced by scopolamine (*p* < 0.001 for 3 mg/kg; *p* < 0.0001 for 10 mg/kg, and 30 mg/kg), and so did donepezil (*p* < 0.0001). However, there were no differences in the activity of BuChE among these groups, as shown in Figure 7B. The result of ACh levels is given in Figure 7C. Compared with the control group, the ACh level of the model group decreased (*p* < 0.05). Donepezil and DL0410 (10 mg/kg, 30 mg/kg) could significantly increase the ACh level in comparison with the model group (*p* < 0.05 for all).

### 2.6. DL0410 Could Interact with the Dual Binding Site of AChE

**Figure 8:**
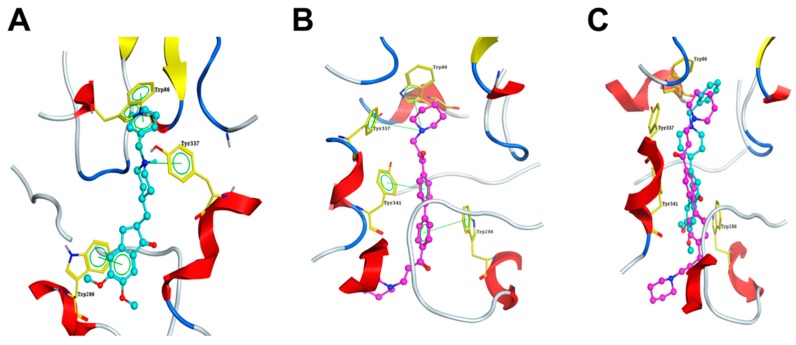
The binding mode of DL0410 and donepezil in the active site of AChE (PDBID: 4EY7). Compounds are displayed in ball-stick model, with carbon of donepezil in cyan (**A**) and carbon of DL0410 in purple (**B**); and the binding residues are shown in stick model with carbon in yellow; (**C**) The superposition of DL0410 and donepezil in the the active site of AChE. Nitrogen is shown in blue, and oxygen is in red.

Regarding to the inhibition of DL0410 on AChE activity in vivo and in vitro [13], we further investigated the binding mode of DL0410 in the active site of AChE, with donepezil as a reference. Donepezil is the only AChE inhibitor binding with a dual binding site (the catalytic active site (CAS), and the peripheral anionic site (PAS)) in AChE. When donepezil was docked into the active site of AChE, it extended from the anionic subsite of the active site to the peripheral anionic site. As shown in Figure 8A, the benzyl ring interacted with the five-and six-membered ring of the Trp86 indole through π-π stacking, and the charged nitrogen of the piperidine ring displayed a cation-π interaction with the phenyl ring of Tyr337 and Trp86 indoles in the CAS. In the PAS, the indanone ring stacked on the indole rings of Trp286 by π-π interaction.

Similarly, the symmetrical structure of DL0410 interacted with CAS and PAS of AChE, respectively. As shown in Figure 8B, the charged nitrogen of the piperidine ring made a cation-π interaction with the indole ring of Trp86 and the phenyl ring of Tyr337 in the CAS, and two phenyl rings displayed a π-π interaction with Tyr341 and Trp286 in the PAS, respectively. The interaction between DL0410 and AChE was similar to that of donepezil. When two docking configurations were overlaid, apart from the piperidine ring of DL0410 outside the PAS, the other part resembled the posture of donepezil, as shown in Figure 8C.

## 3. Discussion

Acetylcholine is widely spread in the central nervous system, and plays a vital role in the development of cortical regulation of cerebral blood flow and sleep cycle, and participating in the process of learning and memory [4]. It establishes the synaptic connection among neurons, which is the basis of cognition and memory [5]. A gradual decline of cholinergic function in aging is accompanied by axon and dendrite degeneration [14,15], leading to mild cognitive impairment, while cholinergic neuron loss predominantly occurs in pathological aging, such as AD, which further impairs synaptic contacts, and leads to irreversible cognitive decline [16,17]. A vast number of experimental studies suggested an association of cholinergic deficits with cognitive impairments [5], which led to the formation of the cholinergic hypothesis in AD. Therefore, AChE inhibitors, ameliorating the hypofunction of the cholinergic system, have been focused on the therapeutic treatment of dementia, especially AD [18,19]. The scopolamine-induced amnesia effect in mice could imitate the deficiency of the cholinergic system, and is used extensively to assess the anti-amnesia effect of drugs. In the present study, the ameliorating effect of DL0410 on the cognitive impairment induced by scopolamine was evaluated, and functions of the cholinergic system, including the activity of AChE and BuChE and ACh levels, and the binding mode of DL0410 in the active site of AChE, were also characterized to further illustrate its potential mechanism.

In the locomotor activity test, we found that the motor function of mice administered scopolamine and drugs (donepezil and DL0410) was not affected, and the difference in the following behavioral tests could be attributed to that in memory and cognition. The Morris water maze (MWM) is a main task that measures spatial memory responsible for recording information about the environment and spatial orientation, and dependent on hippocampus function [20]. Its goal is finding an escape by using environment cues, which rely on the capability to retrieve and retain acquired information [21]. In the navigation trial of the Morris water maze, escape latency and swimming distance of mice were significantly increased by scopolamine, while DL0410 (3 mg/kg and 10 mg/kg) could notably reduce the escape latency and swimming distance of mice. In the probe trial, the latency to cross the platform position and times of crossing the platform position were significantly extended and reduced, respectively. DL0410 could shorten the latency to cross the platform (by 3 mg/kg, 10 mg/kg, and 30 mg/kg) and increase entry times onto the platform (by 10 mg/kg). In the passive avoidance tasks, mice learn to avoid the aversive stimulus, the fear of which enhances the memory encoding by hippocampus [22]. In the step-through and step-down test, scopolamine shortened the latency to suffer from electric shock, and increased error times that mice received an electric shock in the retention phase. DL0410 could significantly reduce the error times, while having no effect on the shorted latency. In sum, DL0410 could improve the memory and cognition function impaired by scopolamine, but in an inverted-U-shape dose-response manner. That is to say, DL0410 performed best at the concentration of 10 mg/kg, which could catch up with donepezil at 3 mg/kg.

In in vitro experiments, DL0410 was found to be a dual inhibitor of AChE and BuChE. In the previous study, we found that AChE activity in the brain was not influenced in the APP/PS1 mice, and DL0410 slightly inhibited the AChE, but without significant difference; however, the BuChE activity in plasma was elevated notably, and DL0410 could significantly reduce BuChE activity [11]. In the scopolamine-induced amnesia mouse model, we found that AChE activity in the model group was significantly increased compared with the control group, which was consistent with the literature [23,24], and DL0410 (3 mg/kg, 10 mg/kg, and 30 mg/kg) suppressed AChE significantly. However, the activity of BuChE was not altered among these groups. In addition, the ACh level in the model group was significantly lower than the control group, and DL0410 (10 mg/kg and 30 mg/kg) could increase the ACh level. Thus, in the present study, the increased ACh level in a dose-dependent manner, could be perfectly attributed to the inhibition of AChE, without BuChE being involved.

However, this phenomenon disagreed with the inverted-U-shaped dose-response relationship in the behavioral test. DL0410 at 30 mg/kg increased the ACh in the synapse cleft to the highest level, but could not improve the cognitive function to the best level. Similarly, donepezil and 10 mg/kg DL0410 enhanced the memory to the optimal condition without the highest level of ACh. On one hand, it has been reported that high levels of ACh could enhance the encoding of information, which leads to a response to the stimulus in the training phase, but suppresses the excitatory feedback which mediates consolidation and retrieval after training [25]. On the other hand, there exists five subtypes of muscarinic receptors (M1, M2, M3, M4, M5) in the hippocampus [26]. The M2 and M4 receptors are predominantly located in the presynaptic membrane, acting as autoreceptors to inhibit the release of Ach [27,28]. High levels of ACh might act on the autoreceptors to inhibit its synthesis and release in the presynaptic neurons, which subsequently affected the acquisition of information. Therefore, there must be an optimized level of ACh in the synapse cleft of hippocampus, leading the learning and memory to the maximized extent, which could explain the disagreement mentioned above.

In the exploration of the binding mode, DL0410 interacted with CAS and PAS of AChE in a similar manner with donepezil, which is the only dual-binding-site AChE inhibitor [29,30]. DL0410 extended the entire active-site gorge of AChE, making a cation-π and π-π interaction with the indole ring of Trp86, and the phenyl rings of Tyr337 and Tyr 341 in the CAS, and a π-π interaction with indole rings of Trp286 in the PAS. When DL0410 was superposed on the donepezil, the parts inside the gorge were similar, but the piperidine ring of DL0410 was outside the PAS. With the PAS completely occupied by DL0410, the entrance of ACh and Aβ aggregation induced by PAS may be blocked better. This facilitates our understanding that DL0410 might inhibit AChE via direct binding in its CAS and PAS simultaneously [31].

The deficits in the cholinergic system are accompanied by the formation of Aβ plaque in the process of AD. Aβ might impair the cholinergic neurons by disturbing the energy metabolism [32], affecting the function of proteome, promoting apoptosis [33], acting on the nAChR [34,35], and so on. Furthermore, the degenerated cholinergic system could lead to Aβ aggregation [36]. It has been found that AChE co-located with Aβ plaque in the brains of AD patients and bound with Aβ via its peripheral site inducing Aβ fibrillogenesis [37,38]. However, it is still a matter to debate whether the impaired cholinergic system is the primary or secondary event to Aβ. It is interesting that a series of dual-binding-site AChE inhibitors have been found to inhibit AChE-induced Aβ aggregation, which could break the vicious cycle and modify the course of disease [39,40,41,42]. The improvements of DL0410 on the cognitive function have been proved in a series of experiments, and its effect on AChE inhibition has already been verified in this study. However, as a dual-binding-site AChE inhibitor, the molecular mechanism of DL0410 on the suppression of Aβ aggregation deserves more investigation, which could help better understand its therapeutic effects.

In conclusion, DL0410 improved the memory and cognitive function in the scopolamine-induced amnesic mouse model, and AChE inhibition might be an important mechanism. Further research on DL0410 anti-amnesia should be done to provide more evidence for its therapeutic effect on dementia.

## 4. Materials and Methods

### 4.1. Drugs and Reagents

DL0410 (≥98% by HPLC) was synthesized by the Institute of Materia Medica, Chinese Academy of Medical Sciences (Beijing, China). Donepezil was purchased from Shandong Jinan Dexinjia Bio-technology Limited Company (Shandong, China). Scopolamine was purchased from TCI Development Co., Ltd. (Shanghai, China). Amplex^®^ Red Acetylcholine/Acetylcholinesterase Assay Kit was purchased from Invitrogen (Carlsbad, CA, USA). S-butyrylthiocholine chloride (BuSCh), 5,5’-dithiobis(2-nitrobenzoic acid) (DTNB) was purchased from Sigma Aldrich (St. Louis, MO, USA).

### 4.2. Animals and Treatments

One hundred male ICR mice (18–20 g, eight-week-old) were purchased from Vital River Lab Animal Technology Co., Ltd. (Beijing, China). Five mice were housed in a cage under a controlled environment with a temperature of 23 ± 1 °C and humidity 50% ± 10%. The environment was under a 12 h light/dark cycle, with light on from 08:00–20:00. Mice had free access to the food and water.

**Figure 9:**
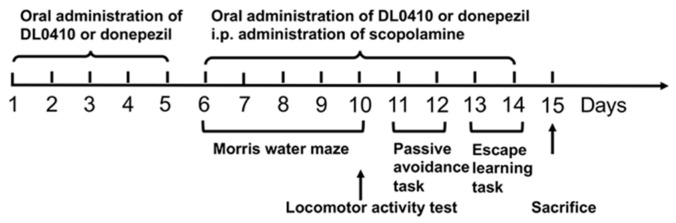
The schematic overview of the experiment.

Mice were allowed to adapt to the new environment for five days before experiments. Mice that reacted to electric shock too sensitively or without response were excluded. Seventy-two mice were selected and randomly divided into six groups, with 12 mice per group. Mice in the control group were treated with saline intraperitoneally (i.p.) and orally administered with water; mice in the model group were treated with scopolamine (1 mg/kg, i.p.) and orally administered with water; mice in the donepezil 3 mg/kg group treated with scopolamine (1 mg/kg, i.p.) and orally administered with donepezil (3 mg/kg); mice in the other three groups were treated with scopolamine (1 mg/kg, i.p.) and orally administered with DL0410 (3, 10, and 30 mg/kg, respectively). Scopolamine was dissolved in sterile saline, and donepezil and DL0410 dissolved in distilled water. They were given at a volume of 0.1 mL/10 g. Donepezil and DL0410 were administered 30 min before injection of scopolamine. All behavioral tests were conducted 30 min after the injection of scopolamine. DL0410 or donepezil was administrated for the 15 days of the whole experiment and once per day, and scopolamine was administered from the sixth day to the end of experiment and once per day. The arrangement of our study is shown in Figure 9.

Animal treatment and maintenance were performed according to the guidelines established by the National Institutes of Health for the care and use of laboratory animals, and were approved by the Animal Care Committee of Peking Union Medical College and Chinese Academy of Medical Sciences.

### 4.3. Locomotor Activity Test

A locomotor activity test was conducted on the fifth day of the Morris water maze test, immediately before the probe trial. The locomotor apparatus consists of a round box surrounded by six infrared probes. A mouse was put into the box with 1 min adaption, then locomotor activity for five minutes was recorded. When the mouse moved in the box, infrared probes could detect the signal and denoted the movement as one time. The locomotor activity was the accumulation of detected signals.

### 4.4. Morris Water Maze (MWM) Test

The apparatus is composed of a black circular pool with diameter of 100 cm and a height of 40 cm. The pool was divided into four quadrants, and the platform (diameter of 8 cm, and height of 20 cm) was fixed in the center of the first quadrant. The pool was filled with water (23 ± 1 °C), with the platform hidden 1 cm blow the water surface.

The Morris water maze test lasted for five days. During the first four days (navigation trial), each mouse was allowed to swim for 60 s twice. The mice were placed into the water facing the wall at the middle of the third quadrant for the first time, and at the middle of the second quadrant for the second time. During the period of swimming, the escape latency and distance were recorded. If a mouse found and stood on the platform, the recording system stopped and the mouse was allowed to stay on the platform for 15 s. If a mouse could not find the platform within 60 s, it was guided to the platform and placed on the platform for 15 s. On the fifth day (probe trial), the platform was removed from the maze, and mice were allowed to swim for 120 s. The swimming paths were recorded and analyzed. The performance of the fourth and the fifth day was regarded as the final result of the navigation trial and the probe trial, and used to assess the spatial memory [43,44].

### 4.5. Passive Avoidance Task

There was a transparent chamber (11.5 cm × 9.5 cm × 11 cm), a black chamber (23.5 cm × 9.5 cm × 11 cm), and a door between them in the apparatus. The black chamber was equipped with a metal floor, which was connected to electricity (36 V, 1.5 mA). In the acquisition phase, mice were put into the transparent chamber illuminated by a lamp, with their back to the black chamber. Firstly, the door was closed, and mice were allowed to accommodate themselves for 1 min. Then the power was turned on, and the door was opened. When mice stepped into the black chamber, they would suffer from electric shock, which was recorded as one error time. This phase lasted for 5 min, and if mice did not step into the black chamber and suffer from electric shock, at the end of the phase mice were gently forced into the black chamber to obtain this experience. Twenty-four hours later, a retention test was conducted, and mice went through the same process. During these two phases, latency (the time mice firstly step into the black chamber) and error times (total times of stepping into the black chamber) were recorded [12].

### 4.6. Escape Learning Task

An escape learning task was conducted in a box (26 cm × 17 cm × 9 cm) with an insulated columnar platform fixed on the metal floor. The floor was connected to electricity (36 V, 1.5 mA).

In the acquisition phase, mice were put into the box to accommodate for 1 min. Then mice were put on the platform, and the power was turned on. When mice stepped down to the metal floor, they would suffer from electric shock, which was recorded as one error time. This phase lasted for 5 min, and if mice did not place their paws on the metal floor and suffer from electric shock, then mice should be gently forced to step down at the end. A retention test was conducted 24 h later, and the same process was repeated. During these two phases, latency (the time mice first step down to the metal floor) and error times (total times of stepping down to the metal floor) were recorded [12].

### 4.7. Preparation of Mouse Brain Tissue

Twenty-four hours after the behavior tests, mice were sacrificed and the bilateral hippocampus was dissected and stored in −80 °C. Before the assay, the hippocampus was homogenized by Omni bead ruptor in five volumes of 0.01 M PBS (137 mM NaCl, 2.7 mM KCl, 10 mM Na_2_HPO_4_, 2 mM KH_2_PO_4_), and then homogenates were centrifuged at 12,000× *g* for 30 min at 4 °C. The supernatant was used for measurement of AChE activity, BuChE activity, and Ach level. The protein concentration was determined by the BCA protein assay kit (Applygen Technology Inc., Beijing, China).

### 4.8. Acetylcholinesterase (AChE) Activity Assay

The measurement of AChE activity was conducted following the instruction of Amplex^®^ Red Acetylcholine/Acetylcholinesterase Assay Kit. This assay was conducted in a 96-well plate, and each reaction contained a 100 μL sample, 50 μM acetylcholine, 200 μM Amplex Red reagent, 0.1 U/mL choline oxidase, and 1 U/mL horseradish peroxidase (HRP) in reaction buffer. The reaction systems were incubated at room temperature for 20 min with protection from light, and then fluorescence intensity was measured at the excitation wavelength of 560 nm and the emission wavelength of 580 nm [45].

### 4.9. Butyrylcholinesterase (BuChE) Activity Assay

The BuChE activity was determined following the method of Ellman with some modification [8,46]. BuChE could hydrolyze BuSCh to produce choline iodide that reacts with DNTB, with the production of TNB. TNB could be quantitated by colorimetry, which indicates the BuChE activity. This assay was conducted in a 96-well plate, and each reaction system was composed of a 40 μL sample, 70 μL BuSCh (7.5 mM), and 80 μL DTNB (0.25 mg/mL). The OD412 was measured after incubation for 60 min at 37 °C.

### 4.10. Acetylcholine Assay

The detection of ACh activity was conducted in accordance with the protocol of the Amplex^®^ Red Acetylcholine/Acetylcholinesterase Assay Kit. Each reaction contained a 100 μL sample, 0.5 U/mL acetylcholinesterase, 200 μM Amplex Red reagent, 0.1 U/mL choline oxidase, and 1 U/mL HRP in reaction buffer. The reaction systems were incubated at room temperature for 30 min with protection from light, and then fluorescence intensity was measured at an excitation wavelength of 560 nm and an emission wavelength of 580 nm [24].

### 4.11. Molecular Docking

The crystal structure of AChE in complex with donepezil (PDB ID: 4EY7) and BuChE in complex with tacrine (PDB ID: 4BDS) was downloaded from PDB [47]. Molecular docking was conducted in MOE 2009 (Molecule Operating Environment) [48]. Before docking, DL0410 was prepared with washing and energy minimization in the MMFF94 force field, and receptor was preceded with removing water and adding hydrogen atoms and partial charge. The active site pocket was defined by the ligand. The detailed parameters set in docking were the default [49].

### 4.12. Statistics

Data were expressed as the mean ± SEM, and were evaluated with one-way ANOVA by Dunnett’s test. Statistical analysis was conducted in GraphPad PRISM software (San Diego, CA, USA), and *p* < 0.05 was considered to be significant.
